# Hospital Election

**Published:** 1867-01

**Authors:** 


Hospital Election.—The annual election of the Buffalo General Hospital was held yesterday afternoon, the following Trustees being elected: Charles E. Clarke, Henry Martin, R. D. Sherman, S. G. Cornell, S. G. Austin, Geo. S. Wardwell, Jason Parker, Thos. F. Rochester, O. G. Steele. The annual meeting ot the Association was held immediately after the closing of the polls, when the annual reports of the Treasurer and Superintendent were read.

